# Genotypes truncating the intracellular tail of human pre-TCRα: From amorphic to isomorphic

**DOI:** 10.70962/jhi.20250155

**Published:** 2025-11-19

**Authors:** Marie Materna, Christian Beetz, Christian A. Ganoza, Caroline Deswarte, Lucien Pereira Estrela, Nima Parvaneh, Jean-Laurent Casanova, Vivien Béziat

**Affiliations:** 1 Laboratory of Human Genetics of Infectious Diseases, INSERM U1163, Necker Hospital for Sick Children, Paris, France; 2 Paris Cité University, Imagine Institute, Paris, France; 3 Centogene GmbH, Rostock, Germany; 4 St. Giles Laboratory of Human Genetics of Infectious Diseases, Rockefeller Branch, The Rockefeller University, New York, NY, USA; 5 Division of Allergy and Clinical Immunology, Department of Pediatrics, Tehran University of Medical Science, Tehran, Iran; 6 Department of Pediatrics, Necker Hospital for Sick Children, AP-HP, Paris, France; 7 Howard Hughes Medical Institute, New York, NY, USA

## Abstract

Using large genomic datasets, we identified six individuals homozygous for intracellular tail truncating PTCRA variants. Functional assays revealed two hypomorphic and one neutral alleles, demonstrating that most intracellular truncations retain pre-TCRα activity, establishing the tail as largely dispensable for pre-TCR assembly.

## Teaser

We identified six individuals with homozygous truncating variants in the pre-TCRα intracellular tail, revealing hypomorphic and neutral alleles. Functional assays show the tail is largely dispensable for pre-TCR expression and highlight the need for experimental validation of such variants.

αβ T lymphocytes constitute one of the three cellular lineages of adaptive immunity (1). They differentiate in the thymus through discrete developmental stages involving somatic rearrangement of the T cell receptor (TCR) gene loci. The development of αβ T cells begins with rearrangement of the *TRB* locus, which encodes the TCR-β chain. This step, known as β-selection, is dependent on the invariant pre-TCRα chain, which mediates the surface expression of a productively rearranged TCR-β. Successful β-selection promotes thymocyte survival, proliferation, and progression to later stages of differentiation. We recently described the first human patients with complete or partial pre-TCRα deficiency (2). Complete pre-TCRα deficiency results in profound neonatal αβ T cell lymphopenia and clinical features with a later onset, including infections, autoimmunity, and lymphoproliferation. By contrast, partial pre-TCRα deficiency results in high naïve γδ T cell counts without significant αβ T cell lymphopenia but with a predisposition to autoimmunity. All pathogenic variants reported to date affect the extracellular or transmembrane domains, thereby preventing expression of the pre-TCR at the cell surface. No homozygous truncating pre-TCRα variants affecting the intracellular tail (ICT) have been reported in patients or even in large population databases, such as gnomAD v4.1. Such variants are unlikely to induce mRNA decay because the ICT is encoded by the last exon. However, previous studies suggest that a “tailless” p.Thr168* allele is loss-of-function (3). This biochemical allele has not, to our knowledge, been reported in humans (e.g., in gnomAD v4.1). However, it raises the possibility that biallelic truncating variants in the ICT may underlie pre-TCRα deficiency and the corresponding immunological and clinical phenotypes.

**Figure 1.**
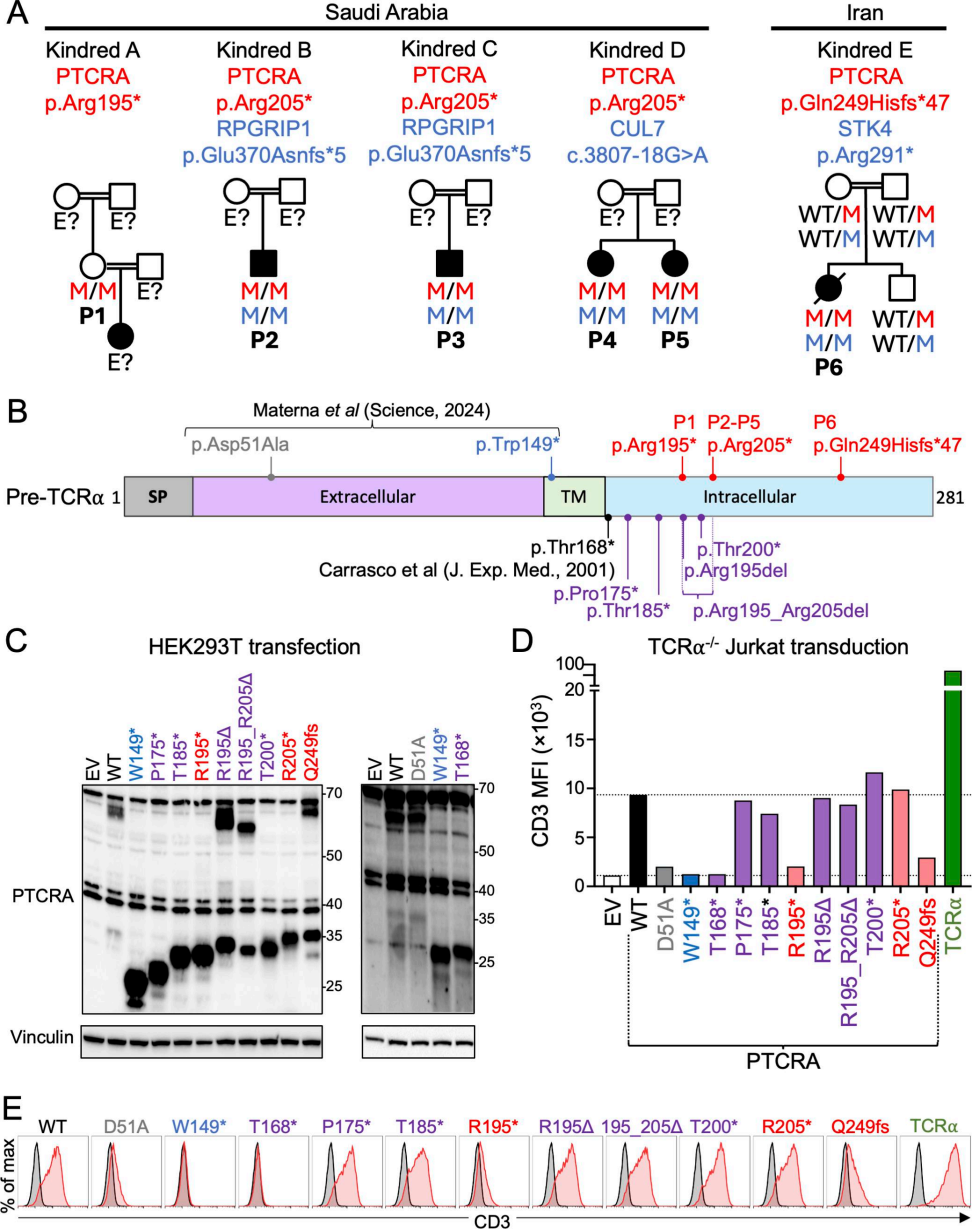
**Functional characterization of pre-TCRα variants truncating the intracellular domain. (A)** Pedigrees of the five kindreds carrying homozygous premature stop or frameshift variants in the intracellular domain of the pre-TCRα chain encoded by *PTCRA* (NM_138296.3). P2 and P3 were diagnosed with RPGRIP1 deficiency (NM_020366.4: c.1107del; p.Glu370Asnfs*5). P4 and P5 had a suspected diagnosis of CUL7 deficiency (NM_014780.5: c.3807-18G>A; splice AI Δ score for an acceptor gain = 0.53). P6 was diagnosed with STK4 deficiency (NM_006282.5: c.871C>T; p.Arg291*). The PTCRA variants appear in red, whereas the variants of other genes appear in blue. M: mutant allele, E?: unknown genotype **(B)** Schematic representation of the PTCRA isoform A protein (2), indicating the location of the variants studied. The variants from the patients appear in red. The artificial variants created in this study appear in purple. SP, signal peptide; TM, transmembrane domain. **(C)** HEK293T cells were transfected with an empty vector (EV), or with plasmids encoding the WT or indicated *PTCRA* isoform A variants. Total protein extracts were analyzed by immunoblotting with an antibody against pre-TCRα or vinculin (loading control). **(D and E)** TCRα-deficient Jurkat cells were transduced with EV or with plasmids encoding WT or mutant pre-TCRα isoform A. CD3ε surface expression was assessed by flow cytometry. **(D)** Bar graphs showing mean fluorescence intensity (MFI) for CD3ε. **(E)** Representative flow cytometry histograms. In each histogram, the trace of cells transduced with the EV is shown in grey, and the trace of cells transduced with the indicated variant is shown in red. All results are representative of three independent experiments. The methods used in C–E have been described in detail elsewhere (2). Source data are available for this figure: SourceData F1.

Using a reverse genetics approach, we screened our in-house database (∼30,000 whole-exome sequencing[WES]/whole-genome sequencing [WGS]) and the Centogene database (∼185,000 WES/WGS) (Fig. 1 A), identifying six individuals homozygous for variants truncating the ICT of pre-TCRα (NM_138296.3, encoding isoform A^2^). Patient 1 (P1) was homozygous for c.583C>T, introducing a premature stop codon at Arg195 (p.Arg195*). This variant appeared 1,088 times in the heterozygous state in gnomAD v4.1 (allele frequency 0.0006995) and was most frequent in Europeans (0.0009198; 1,057 heterozygotes). Patients 2 (P2), 3 (P3), 4 (P4), and 5 (P5) were homozygous for c.613C>T, generating a premature stop at Arg205 (p.Arg205*). This variant was reported 33 times in the heterozygous state in gnomAD v4.1 (allele frequency 0.00002122), with the highest frequency in the Middle East (0.0003357). Patient 6 (P6) was homozygous for c.745_746dup, causing a frameshift at Gln249 with the addition of 47 residues (p.Gln249Hisfs*47). Her sister and both parents were heterozygous for this variant. This variant was reported three times in the heterozygous state in gnomAD v4.1 (one European and two Ashkenazi Jewish individuals; allele frequency: 0.000001859). All three variants are predicted to truncate the ICT of the pre-TCRα protein, leading to the loss of the last 87, 77, and 33 amino acids, respectively, at the C terminus of the protein. However, these truncated proteins have intact extracellular and transmembrane domains, suggesting that the truncated proteins may retain full or partial functionality.

All patients were born to consanguineous parents (Fig. 1 A). P1 was an 18-year-old woman from Saudi Arabia. She was the unaffected mother of an infant with a severe neurodevelopmental phenotype, and her detailed health status was unknown. P2 and P3 were unrelated Saudi patients diagnosed with Leber congenital amaurosis 6 (Online Mendelian Inheritance in Man [OMIM] #613826) due to homozygosity for a retinitis pigmentosa GTPase regulator interacting protein 1 (RPGRIP1) variant. At ages 5 and 35 years, respectively, they displayed no unusual immunological features. P4 and P5, 6 mo and 3 years old, respectively, were Saudi sisters presenting with failure to thrive, skin hyperextensibility, and skin and joint hypermobility, but no immunological abnormalities. They were suspected to have 3M syndrome (OMIM #273750) due to homozygosity for a private cullin 7 (CUL7) variant. No biological data were available for P1–P5. P6 was a 19-year-old Iranian woman who received standard neonatal vaccinations, including Bacillus Calmette-Guérin (BCG) and oral polio. In infancy, she developed BCG-itis requiring surgical drainage and experienced recurrent respiratory infections throughout childhood. She developed chronic epidermodysplasia verruciformis-like lesions on her neck and trunk, persistent throughout life, and cytomegalovirus-induced colitis at 5 years. At 7 and 9 years, she had Henoch–Schönlein purpura, followed by noninfectious arthritis of the right knee at 11 years. At 19 years, she developed a fatal plasmablastic lymphoma. She consistently displayed B cell, CD4^+^, and CD8^+^ T cell lymphopenia with normal immunoglobulin levels. In addition to homozygosity for the pre-TCRα (*PTCRA*) variant, she carried a biallelic loss-of-function serine/threonine kinase 4 (*STK4*) variant (c.871C>T; p.Arg291*; OMIM #614868), previously reported in a Bangladeshi patient with recurrent infections (4).

Published data indicate that the ICT of human pre-TCRα is important for pre-TCR formation. A “tailless” pre-TCRα construct (p.Thr168*), lacking the entire ICT, was previously shown to fail to stabilize CD3 expression at the surface of JR3.11 TCRα-deficient Jurkat cells (3). We therefore hypothesized that the PTCRA variants p.Gln249Hisfs*47, p.Arg195*, and p.Arg205* might impair pre-TCRα function. HEK293T cells were transfected with plasmids encoding the WT or one of these three mutant PTCRA alleles, or with several controls: the hypomorphic p.Asp51Ala (2), the loss-of-function p.Trp149* (lacking the transmembrane domain) (2), the tailless loss-of-function p.Thr168* (3), and artificial constructs (p.Pro175*, p.Thr185*, p.Thr200*, p.Arg195del, and p.Arg195_Arg205del) (Fig. 1 B). Whole-cell protein extracts were analyzed by western blot using a polyclonal antibody against the N terminus of pre-TCRα (Fig. 1 C). As reported (2), two bands were observed for the WT and p.Asp51Ala isoforms: one at ∼35 kDa—corresponding to the predicted molecular weight (MW)—and another at ∼70 kDa, likely corresponding to a glycosylated form (2). All variants introducing premature stop codons lost the higher MW band and produced a truncated lower band. The small deletions (p.Arg195del and p.Arg195_Arg205del) yielded two bands with MW similar to or slightly below WT. The p.Gln249Hisfs*47 variant produced two bands slightly higher than WT, consistent with the addition of 14 amino acids (293 vs. 281 residues). Notably, a high MW band was preserved only in constructs retaining amino acids 205–249 (WT, p.Arg195del, p.Arg195_Arg205del, and p.Gln249Hisfs*47), suggesting this region is critical for posttranslational modifications in HEK293T cells and warrants further investigation in future studies.

We next assessed the ability of pre-TCRα mutants to stabilize pre-TCR expression at the cell surface. We transduced a TCRα-deficient Jurkat cell line with the *PTCRA* alleles and assessed the ability of the proteins encoded by these alleles to stabilize CD3 expression at the cell surface (Fig. 1, D and E). A TCRα construct resulting in high levels of surface CD3 expression was included as a positive control. As expected (2), WT pre-TCRα stabilized surface CD3 at intermediate levels. In contrast, the p.Trp149* and tailless p.Thr168* mutants failed to stabilize CD3, whereas p.Asp51Ala was severely hypomorphic, supporting only weak CD3 expression (2, 3). The p.Arg195* and p.Gln249Hisfs*47 mutants were also hypomorphic, stabilizing CD3 at levels similar to p.Asp51Ala. By contrast, the other intracellularly truncated proteins (p.Pro175*, p.Thr185*, p.Thr200*, and p.Arg205*) and the in-frame deletions (p.Arg195del and p.Arg195_Arg205del) were functionally neutral, supporting normal CD3 stabilization. Collectively, these results indicate that none of the ICT mutants—except the tailless p.Thr168* variant—caused a complete loss-of-function. The ability of the p.Pro175* mutant to support normal pre-TCR expression suggests that retention of as few as seven intracellular amino acids is sufficient for proper pre-TCRα function. However, two more distal truncations, p.Arg195* and p.Gln249Hisfs*47, were consistently hypomorphic. The molecular mechanism underlying the partial loss-of-function of truncations more distal than p.Pro175* remains unclear. It appears disconnected from the posttranslational modification profile observed in the HEK293T cell overexpression system (Fig. 1 C) and should be investigated in future studies.

In conclusion, using a reverse genetics approach, we identified six individuals homozygous for premature stop or frameshift *PTCRA* variants truncating the ICT of pre-TCRα. Two variants, each found in one patient, were hypomorphic (p.Arg195* and p.Gln249Hisfs*47), whereas another, found in four patients, was functionally neutral (p.Arg205*). Hypomorphic pre-TCRα variants have been associated with normal αβ T cell counts, increased naïve γδ T cell counts, and autoimmunity with incomplete penetrance (2). The lack of biological material prevented deep immunophenotyping or TCR repertoire analyses, representing a limitation of this study. The contribution of partial pre-TCRα deficiency to the clinical presentation of P6, who carried the p.Gln249Hisfs*47 variant, remains uncertain due to concomitant *STK4* deficiency. P1, carrying the p.Arg195* variant, was reported healthy at 18 years, possibly reflecting incomplete penetrance, though no detailed clinical data were available. The neutral effect of the p.Arg205* variant is consistent with the absence of apparent immunological phenotypes in P2–P5. We also confirmed that the tailless p.Thr168* variant disrupts pre-TCR formation in vitro (3). Moreover, retention of as few as seven additional intracellular amino acids—as in p.Pro175*—was sufficient to permit normal pre-TCR surface expression. Overall, our findings indicate that the ICT is largely dispensable for human pre-TCR assembly, consistent with its poor conservation across mammals (5). Nevertheless, the discovery of two hypomorphic ICT-truncating variants demonstrates that definitive functional classification requires in vitro validation. Homozygous *PTCRA* variants should be tested experimentally, even if predicted loss-of-function and even if located in the exon encoding the ICT. These findings are of potential interest to clinicians and diagnostic laboratories likely to encounter *PTCRA* variants in patients.

## Supplementary Material

SourceData F1 is the source file for Fig. 1.

## Data Availability

The data (Fig. 1, C–E) are available from the corresponding author upon reasonable request.
