# Supplementary figures and images for: Genotypes truncating the intracellular tail of human pre-TCRα: From amorphic to isomorphic
Source: J Hum Immun. 2025 Nov 19;2(1):e20250155. doi: 10.70962/jhi.20250155 (PMC12842982; doi:10.70962/jhi.20250155)

Figure 1C - Left panel

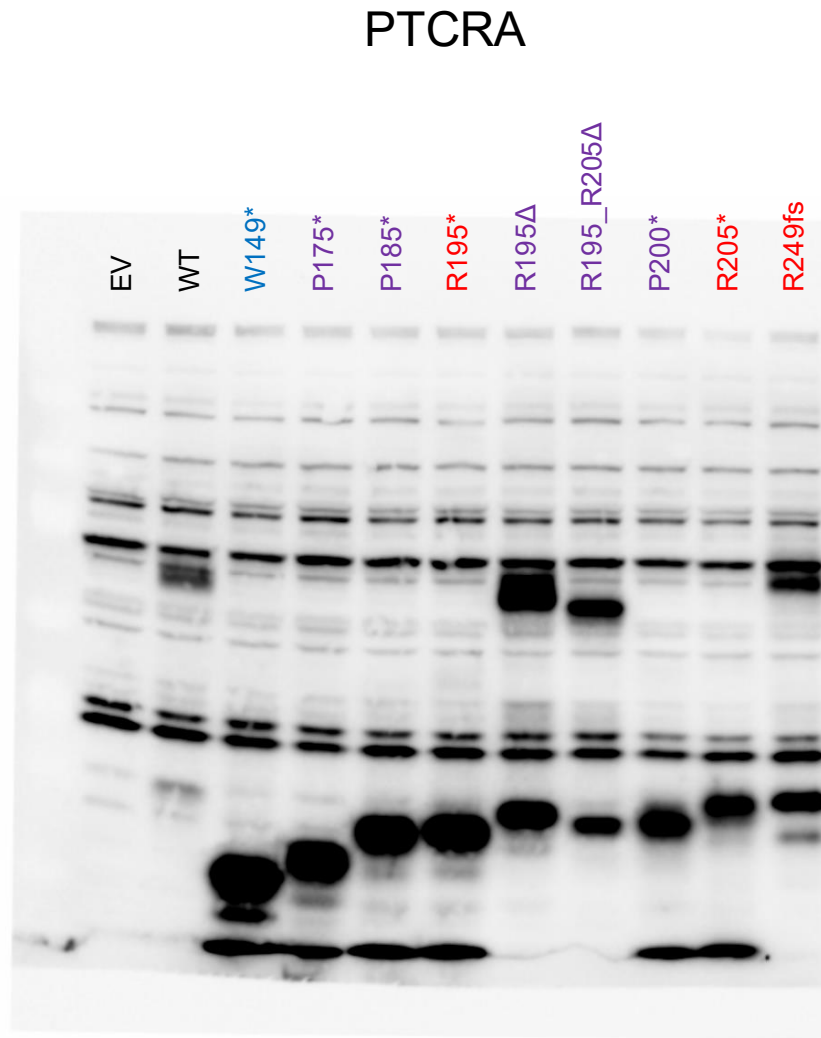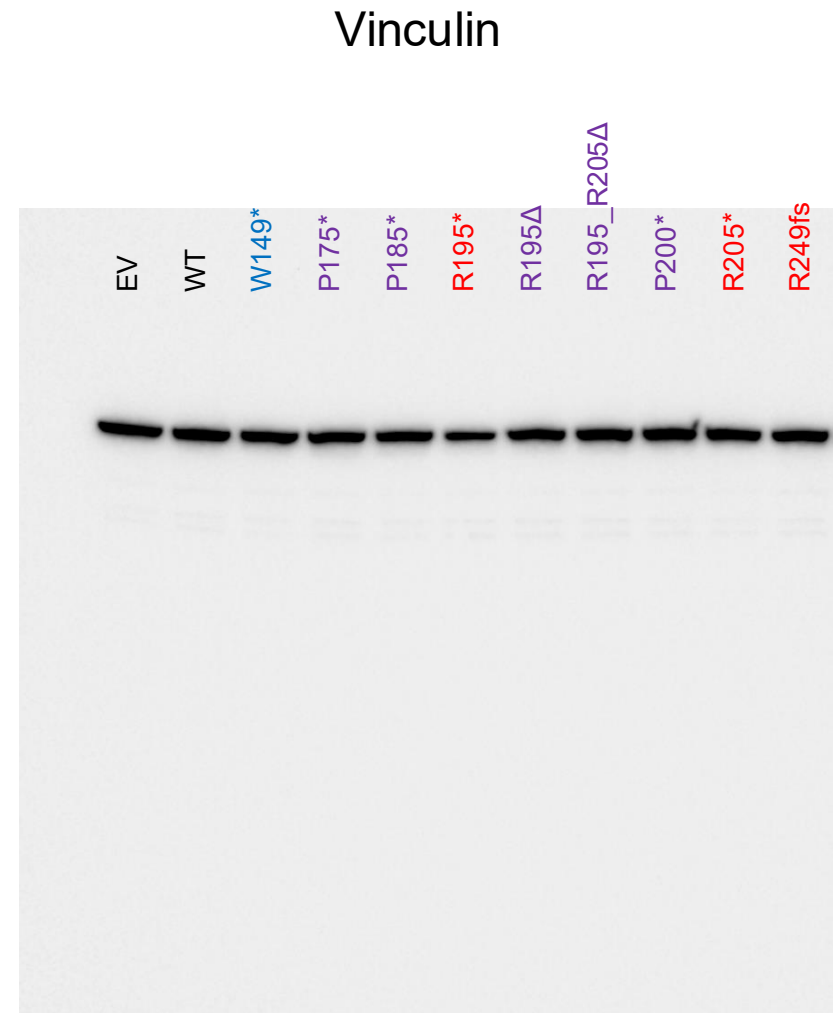

Figure 1C - Right panel

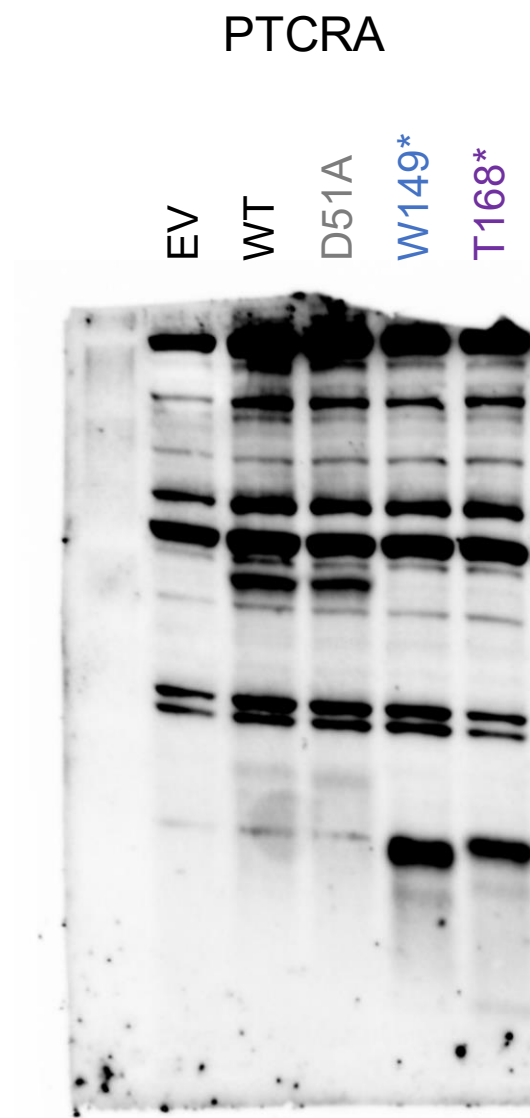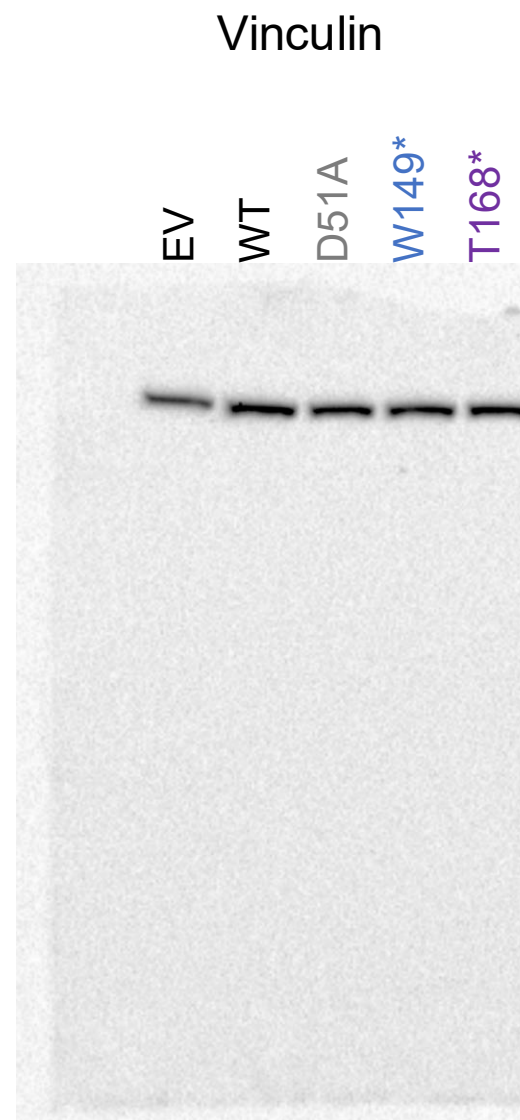

Supplement: SourceData F1 — is the source file for Fig. 1. [file jhi_20250155_sourcedataf1.pdf]
